# Supplementary material for: The Interest of a Systematic Toxicological Analysis Combined with Forensic Advice to Improve the Judicial Investigation and Final Judgment in Drug Facilitated Sexual Assault Cases
Source: Pharmaceuticals (Basel). 2021 May 4;14(5):432. doi: 10.3390/ph14050432 (PMC8147760; doi:10.3390/ph14050432)
Supplement: Supplementary file 1 [file pharmaceuticals-14-00432-s001.zip › pharmaceuticals-1170694-supplementary.pdf]

Table S1 (Supplementary Data): Mass-spectrometric parameters for the detection of neuroleptics in blood and urine

Compound	Precusor ion (m/z)	Product ions (m/z)	Cone Voltage (CV)	Collision Energy (eV)
Sulpride	342,2	112,1	40	30
		214,1	40	40
Tiapride	329,1	132,9	30	50
		177,0	30	50
Olanzapine	313,2	213,0	30	40
		256,1	30	30
Desmethylolanzapine	299,2	213,0	40	40
		256,1	40	30
7-OH-quetiapine	400,2	269,1	40	35
		295,1	40	35
Pimpamperon	376,1	164,9	35	35
		291,0	35	24
Amisulpride	370,2	214,0	30	50
		149,0	30	50
Risperidone	411,3	110,1	35	60
		191,2	35	40
9-OH-Risperidone	427,2	110,0	40	60
		207,1	40	40
Desmethylclozapine	313,2	227,0	30	40
		270,1	30	30
Bemperidol	382,2	122,8	36	52
		164,9	36	34
Clozapine	327,1	84,0	50	30
		192,0	50	40
Quetiapine	384,2	221,0	38	52
		253,0	38	30
Droperidol	380,1	164,9	24	34
		193,9	24	24
Prothipendyl	286,1	213,0	26	38
		241,0	26	24
Haloperidol	376,1	122,9	34	54
		164,9	34	36
Bromperidol	420,1	123,0	35	60
		165,1	35	30
Aripiprazol	448,2	98,1	30	50
		285,1	30	40
Clotiapine	344,1	255,1	34	44
		287,1	34	28
Levomepromazine	329,0	209,9	24	44
		241,9	24	30
Pimozide	462,3	109,1	38	58
		328,2	38	40
Sertindole	441,2	71,0	40	60
		113,0	40	40
Flupentixol	435,1	264,9	42	52
		304,9	42	42
Desmethylolanzapine-d8	307,2	198,1	40	50
Risperidone-d4	415,3	195,1	40	40
9-OH-Risperidone-d4	431,3	211,2	40	40
Clozapine-d4	333,2	274,1	30	40
Quetiapine-d8	392,2	258,1	30	40
Haloperidol-d4	380,2	169,2	35	40
Aripiprazol-d8	456,2	293,1	35	45
